# Dielectric and Ultrasonic Properties of PDMS/TiO_2_ Nanocomposites

**DOI:** 10.3390/polym16050603

**Published:** 2024-02-22

**Authors:** Ieva Vanskeviče, Martynas Kinka, Jūras Banys, Jan Macutkevič, Sebastien Schaefer, Algirdas Selskis, Vanessa Fierro, Alain Celzard

**Affiliations:** 1 Faculty of Physics, Vilnius University, Sauletekio 9/3, LT-10222 Vilnius, Lithuania; i.kranauskaite@yahoo.com (I.V.); martynas.kinka@ff.vu.lt (M.K.); juras.banys@ff.vu.lt (J.B.); 2 Center for Physical Science and Technology, Sauletekio Ave. 3, LT-10257 Vilnius, Lithuania; algirdas.selskis@ftmc.lt; 3 Institut Jean Lamour—IJL, Université de Lorraine, CNRS, 88000 Épinal, France; sebastien.schaeffer@univ-lorraine.fr (S.S.); vanessa.fierro@univ-lorraine.fr (V.F.); alain.celzard@univ-lorraine.fr (A.C.); 4 Institut Universitaire de France—IUF, 75231 Paris, France

**Keywords:** dielectric permittivity, Vogel–Fulcher law, titanium dioxide

## Abstract

This work presents the dielectric and ultrasonic properties of polydimethylsiloxane (PDMS) nanocomposites filled with titanium dioxide nanoparticles. The dielectric study was performed over a very broad range of frequencies (20 Hz–3 THz). The dielectric permittivity was almost frequency-independent in all the composites at room temperature over the whole range of measurement frequencies, and the dielectric losses were very low under these conditions (less than 2). The dielectric permittivity strongly increases with the nanoparticle concentration according to the Maxwell–Garnet model. Therefore, the investigated composites are suitable for various flexible electronic applications, particularly in the microwave and terahertz frequency ranges. Dielectric dispersion and increased attenuation of ultrasonic waves were observed at lower temperatures (below 280 K) due to the relaxation of polymer molecules at the PDMS/TiO_2_ interface and in the polymer matrix. The relaxation time followed the Vogel–Vulcher law, while the freezing temperature increased with the titanium dioxide concentration due to interactions between the polymer molecules and nanoparticles. The significant hysteresis in the ultrasonic properties indicated that titanium dioxide acts as a crystallization center. This is confirmed by the correlation between the hysteresis in the ultrasonic properties and the structure of the composites. The small difference in the activation energy values obtained from the ultrasonic and dielectric investigations is related to the fact that the dielectric dispersion is slightly broader than the Debye-type dielectric dispersion.

## 1. Introduction

Polymeric composites are used often in various areas of human life, particularly as coatings and materials for various components in aviation, marine, automobile and biomedical applications [1]. The superiority of composites is that they have outstanding mechanical properties, optimal thermal resistance, good fire, impact and abrasion resistance and optimal elastic and electromagnetic properties. The interest in polymeric composites has rapidly increased with the development of 3D printing technology [2]. This technology creates new possibilities for developing smart materials in various applications. Many polymers can be used as matrixes for polymeric composite preparation; they can be of the thermoplastic, thermosetting and (or) elastomer type [3]. Examples of matrixes used often for composite preparation are polycarbonates, polyamides, polyesters, phenoplasts, epoxy resins, polyurethanes, polyether ether ketone, poly(butylene succinate-co-adipate) and many others. An important subgroup of polymers used for composite preparation is the elastomers. Elastomers are cross-linked polymers and have the ability to restore the primary shape when stress is removed. Examples of elastomers are polybutadiene, chloroprene rubber, butyl rubber, epichlorohydrin rubber, acrylic rubber, silicon rubber, ethylene-vinyl acetate and many others. Proper composite preparation technology is the main way to obtain improved mechanical, thermal and electromagnetic properties of composites.

Polydimethylsiloxane (PDMS) is one of the most widely used and commercially important low-cost silicon-based elastomers. It is a non-toxic, non-fluorescent, biocompatible, mechanically flexible and reversibly deformable [4] elastomer. It is also optically transparent, gas-permeable [5] and electrically insulating [6]. PDMS is a non-entangled semi-crystalline silicone elastomer that melts at ~233 K, crystallizes at ~183 K and vitrifies upon cooling at ~148 K [7,8]. PDMS can be used in various biological systems, such as biomodels [9], organ-on-a-chip platforms [10], mimicking blood particles in blood analogues [11] and membranes for filtering and pervaporation [12]. The application of PDMS in various areas is mostly driven by the development of soft lithography techniques. One of the factors limiting PDMS for broader applications is its low mechanical strength, modulus of elasticity and dielectric permittivity values. To improve the electrical and mechanical properties of polymeric material, filler particles of micro- or nanometric size are added. The overall properties of the composite can be modified to suit the desired application by choosing the proper filler nature and content [13].

Titanium dioxide (TiO_2_) at the nanoscale is transparent to visible light and strongly absorbs UV [14]. Titanium dioxide nanomaterials are widely used for solar cells, hydrogen storage, photovoltaic and photocatalytic applications, in electrochromic devices and as sensors of various gases [15]. The annual production of titanium dioxide nanoparticles exceeds four million tons per year, and this material has numerous applications as a food, drug and plastic colorant, a component in inks and paints, ceramics, cosmetics and sunscreen, in photovoltaic, photocatalyst and self-cleaning surfaces and in batteries and drug delivery [16]. Various applications of titanium dioxide nanoparticles are possible due to their outstanding photochemical properties and very good biocompatibility [16,17,18]. Titanium dioxide nanoparticles are inexpensive and easily reachable [15]. Titanium dioxide polymorphs are rutile, anatase, akaogilite and brookite [19]. The most frequent form of titanium dioxide that can be found in nature is rutile. It has a large dielectric permittivity value (about 130) [20,21]. Another form of titanium dioxide anastase is rare enough; however, it has great electrical conductivity even at room temperature [20]. Titanium dioxide nanoparticles are a popular filler in various composites [22,23,24,25]. It was demonstrated that these nanoparticles can substantially improve the mechanical properties of epoxy resin composites [22,23]. However, few studies have been published on the dielectric and ultrasonic properties of titanium dioxide-filled silicone composites. These investigations were performed mainly in the low-frequency range and at room temperature or for composites with the anatase form of titanium dioxide nanoparticles [20,21]. It was demonstrated that the orientation of the titanium dioxide nanoparticles in a polymer matrix can have an impact on the composite’s dielectric properties [21]. It is important to note that, although PDMS is one of the most extensively used polymers, the crystallization process of PDMS composites and how filler particles affect its dynamical dielectric properties have not been thoroughly investigated.

PDMS composites are used in microsystem applications, particularly in micro- and nanofluidics [4,26]. The improved properties of nanocomposites are related to changes in the polymer structure and dynamics of the polymer due to the interaction with the filler surface. Flexible composites offer additional solutions for sensor, actuator and transducer applications. Moreover, PDMS composites with carbon nanotubes, onion-like, metallic nanoparticles and other inclusions are widely used for electromagnetic shielding applications [27,28]. PDMS composites can also be used for optical ultrasound generation [29]. PDMS composites were used in many other applications, including as a photocatalyst for water purification [30] and in various multifunctional wearable devices [31], strain-sensitive temperature sensors [32] and others.

Nowadays, electromagnetic waves with frequencies between 1 GHz and several terahertz (microwaves and terahertz radiation) are widely used in various areas of human life, including radars, telecommunications, medicine, drying materials and the preparation of food. This frequency range is of particular interest to researchers and engineers for the development of new devices, electromagnetic radiation sources, passive elements and systems for various applications. Therefore, new materials with unique properties in this frequency range are highly desirable. There can be noticed the complex dielectric permittivity ε* = ε’ − iε’’, an important parameter, which characterizes the linear interaction of electromagnetic waves with non-magnetic materials. The real part of the complex dielectric permittivity (or simply dielectric permittivity) characterizes the stored energy of electromagnetic waves, while the imaginary part of the complex dielectric permittivity (or simply dielectric losses) is related to the dissipated energy of electromagnetic waves. Both dielectric permittivity and dielectric losses are related together according to the Kramers–Kroning relation. For practical applications, materials with big or low dielectric permittivity and (or) dielectric loss values in different frequency ranges are desirable. Composites are materials whose dielectric properties can be easily changed by changing the filler type, concentration, polymer matrix type and composite preparation technology. The rapid development of technologies in this frequency range is restricted, particularly due to insufficient knowledge about the properties of the materials in this frequency range [33]. Specifically, for microwave and terahertz lenses and other passive devices, materials with big dielectric permittivity and low loss values are needed. PDMS composites are attractive materials for flexible terahertz electronics applications [34]. For example, PDMS-based materials can be used for mechanically tunable terahertz metamaterials [35]. However, up to now, investigations of the dielectric properties of PDMS-based composites in the terahertz frequency range are rare enough.

In this study, we present research on PDMS composites filled with titanium dioxide nanoparticles and the influence of the filler concentration on the broadband dielectric and ultrasonic properties of the composites over a wide temperature range.

## 2. Materials and Methods

Titanium dioxide (TiO_2_) nanoparticles with a rutile structure were used to prepare the composites, and PDMS was used as the composite matrix. TiO_2_ with a 100 nm particle size was purchased from US Research Nanomaterials, Inc. (Houston, TX, USA), while the PDMS matrix was purchased from Farnell (Limonest, France) under the reference Sylgard 184 Silicone Elastomer (Dow Corning^®^, Midland, MI, USA).

Prior to synthesis, PDMS was degassed under reduced pressure (4000 Pa, 30 min) at room temperature. For each composite, the corresponding amount of TiO_2_ was dispersed in isopropanol (IPA). The IPA-based suspension containing the TiO_2_ nanoparticles was then sonicated for 3 h in an ultrasonic bath (VWR USC 1200 TH, 600 W, Radnor, PA, USA) and for 5 min using an ultrasonic tip (VWR DIGITAL, 450 W, with a 13 mm diameter probe) at 20% of its maximum power. The suspension was then poured onto the corresponding amount of PDMS and sonicated using the same procedure. To evaporate the IPA, the IPA/TiO_2_/PDMS blend thus obtained was placed overnight in a ventilated oven at 60 °C. The resultant PDMS/TiO_2_ paste was sonicated (ultrasonic bath) once more for 1.5 h. The required amount of curing agent specified by the supplier was then added. The mixture was then gently stirred by hand for 10 min. To remove any air bubbles that may have been trapped during this stage, the final mix was degassed under reduced pressure (4000 Pa, 2 min). Depending on the viscosity of the mixes, the composites were cast or manually pressed into molds before curing. The composites were finally cured for 2 to 3 h at 90 °C in a ventilated oven. All the composites studied were white and not transparent in the optical frequency range. Several filler concentrations were chosen, ranging from 0 to 30 vol.% TiO_2_.

Characterization of the composites was performed by using a variety of experimental techniques, including broadband dielectric spectroscopy and ultrasonic investigations, which are complementary to each other and were carried out over a wide range of frequencies and temperatures. Broadband dielectric spectroscopy is a special investigation method, which allows the investigation of the relaxation processes of various systems over an extremely wide range of characteristic times from 1 ps to 10^6^ s and for investigations to be performed in a very wide frequency range from 10 µHz to several terahertz.

Scanning electron microscopy (SEM) images were obtained using a JSM 6460 LV electron microscope (JEOL, Tokyo, Japan). The complex dielectric permittivity, $\varepsilon^{*}=\varepsilon^{\prime }-i\varepsilon^{\prime \prime }$, was measured as a function of temperature and frequency using an LCR meter (HP4284A, (HP4284A, Hewlett, Spring, TX, USA) over a frequency range from 20 Hz to 1 MHz and a temperature range from 120 to 300 K. Each measurement was started at room temperature; the samples were then cooled down to 120 K at a rate of 1 K/min and then re-heated to room temperature at the same rate. The samples had a height and area of ~2.5 mm and ~25 mm^2^, respectively, and silver paste was used for the electrodes. In the microwave frequency range, from 26 GHz to 37 GHz, the reflectance and transmission of a thin dielectric rod placed inside a waveguide were studied. A custom-built waveguide spectrometer, which includes the generator P2-65 and the scalar network analyzer R2400, was used in this frequency range [36]. The typical rod diameter was several hundred micrometers. In the terahertz frequency range (100 GHz to 3 THz), a time-domain terahertz spectrometer (Ekspla Ltd., Vilnius, Lithuania) was used for the measurements. It is based on a femtosecond laser fiber (wavelength 1 μm, pulse duration less than 150 fs) and a GaBiAs photoconductive terahertz emitter and detector. The main mechanism of terahertz generation in GaBiAs is the optical rectification [37]. The signal-to-noise ratio reached 60 dB at a frequency of 0.5 THz. The complex effective permittivity was calculated using the Fresnel equation [38]:

(1) $$T\left(\omega \right)=\frac{4Nexp[\frac{i\omega \left(N-1\right)d}{c}]}{{\left(N+1\right)}^{2}}\sum_{k=0}^{m}[\frac{N-1}{N+1}\exp(\frac{\mathrm{i\omega Nd}}{c}){]}^{2k}$$

where *N* = *n* − *ik* is the complex reflection index, *d* is the sample thickness, *c* is light velocity and *m* is the number of reflections in the sample. These reflections can be experimentally separated because they are separate pulses in the measured signal so that the value of parameter *m* can be easily determined. The complex dielectric permittivity is related to the complex refraction index via the relation ε* = *N*^2^. The thickness of the samples was varied in order to have the transmission not less than 0.01.

The study of ultrasonic attenuation in PDMS composites containing TiO_2_ nanoparticles at different concentrations was carried out over a temperature range of 150–300 K using an automatic pulse-echo setup described in detail elsewhere [39]. The sample was placed between two ultrasonic waveguides lubricated with silicone oil in order to ensure acoustic contact between the sample and the waveguides. LiNbO_3_ acoustic wave transducers working at 10 MHz frequency were used to transmit and receive the longitudinal acoustic waves. The velocity of the ultrasonic longitudinal wave was calculated from the variation in the delay time in the mechanical system after subtracting the known part of the delay variation in the quartz buffers. Measurements were performed upon heating and cooling at a rate of about 1 K/min. Each measurement began with cooling and, after reaching 150 K, the sample was heated to room temperature. The temperature change within the samples was measured using a Keithley Integra 2700 multimeter (Cleveland, OH, USA) equipped with a thermocouple. For details about the experimental setup, see [40].

## 3. Results

### 3.1. Structure and Dielectric Properties of Composites

**Figure 1:**
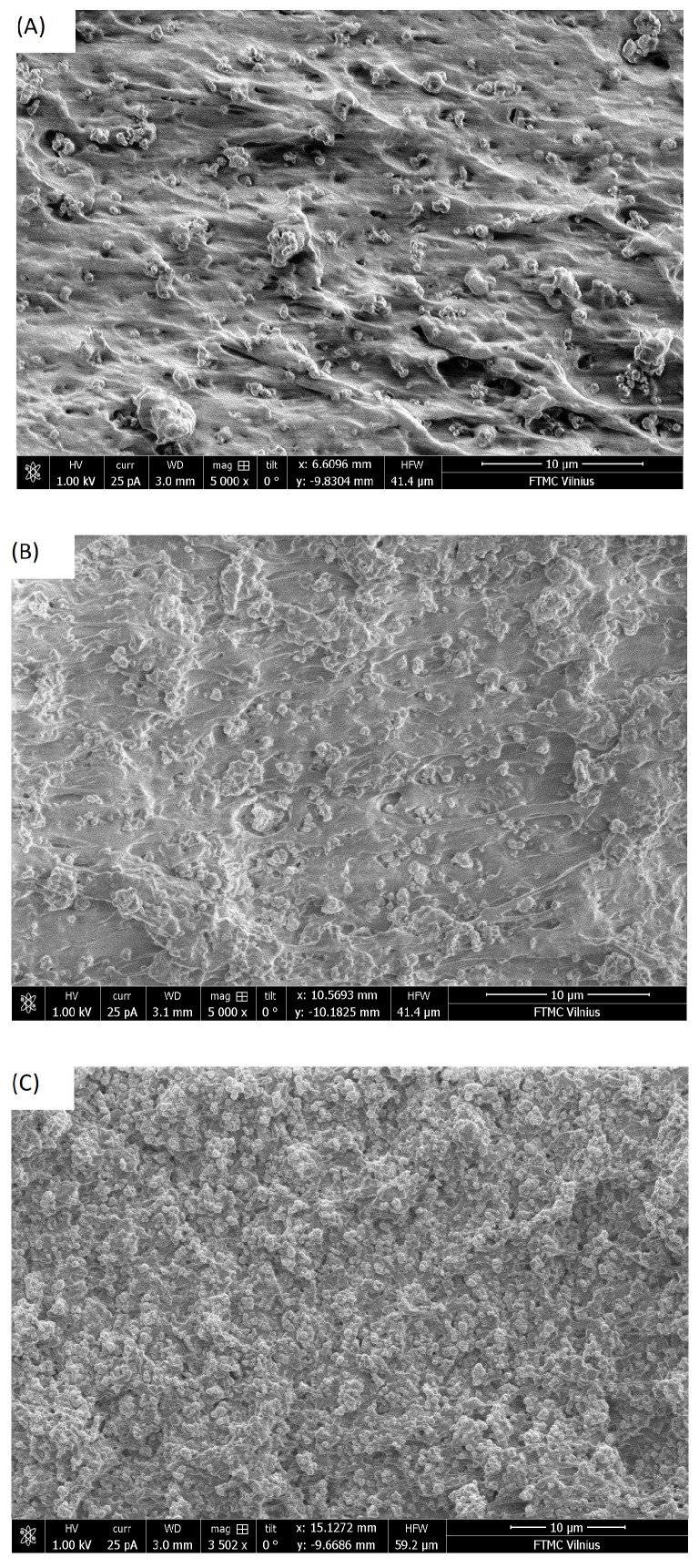
SEM images of PDMS composites filled with TiO_2_ nanoparticles at different concentrations: (**A**) 10 vol.%, (**B**) 20 vol.%, (**C**) 30 vol.%.

SEM images of PDMS-based composite materials filled with TiO_2_ nanoparticles are presented in Figure 1. The TiO_2_ nanoparticles were well dispersed in composites with different filler concentrations, as all the surfaces studied in the composites with the same filler concentrations were similar.

**Figure 2:**
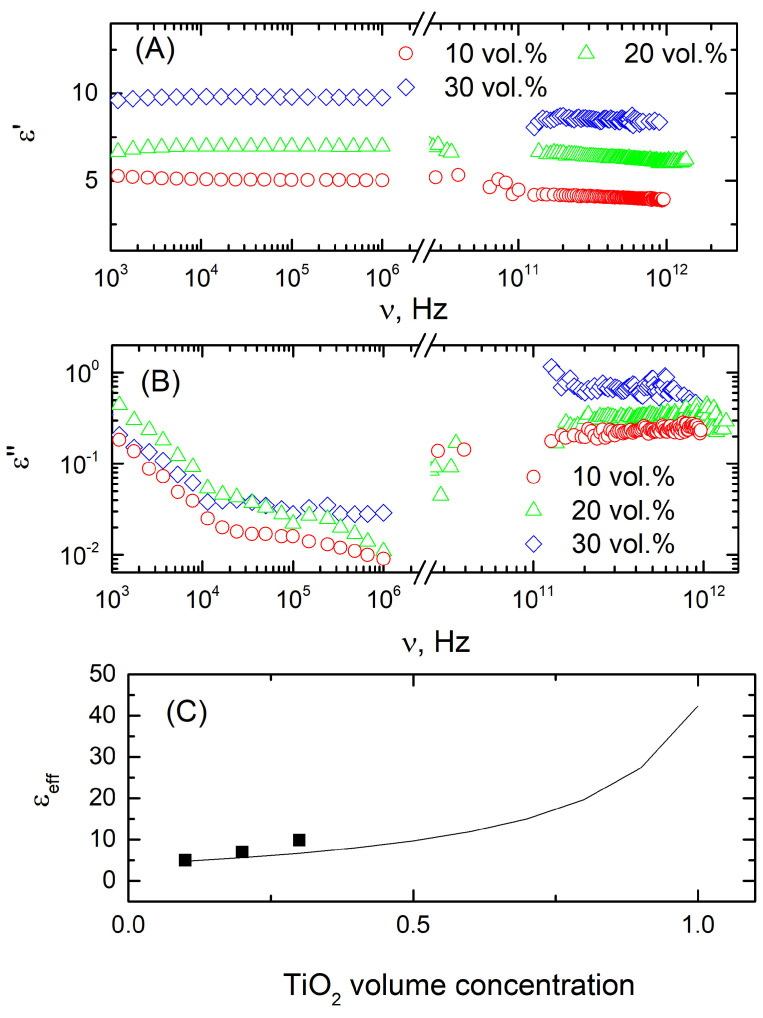
Frequency dependence of the real and imaginary parts of the complex dielectric permittivity of PDMS/TiO_2_ composites at room temperature (**A**,**B**) and concentration dependence of the dielectric permittivity at room temperature and 129 Hz frequency, the solid line is the best fit according to Equation (2) (**C**).

The frequency dependence of the real and imaginary parts of the complex dielectric permittivity of composites with different concentrations of titanium dioxide at room temperature is presented in Figure 2A,B, respectively. The dielectric permittivity is almost constant, while the dielectric losses are very low (below 3), in the frequency range of 1 kHz–1 THz, for different TiO_2_ concentrations. The complex dielectric permittivity is higher for a higher filler concentration, but the dielectric losses remain low, even at higher TiO_2_ concentrations (below 1.3 at 100 GHz). The dielectric losses slightly increase with frequency in the microwave and terahertz frequency ranges; however, their values remain low even above 1 THz frequency (lower than 1.3). Such behavior of dielectric losses is due to the contribution of phonons typical for PDMS and TiO_2_ in infrared and terahertz frequency ranges [41,42]. The small increase in dielectric losses at low frequencies (below 10 kHz) is consistent with Jonsher universal law [43].

The concentration dependence of the composites can be approximated by effective medium theory, for example, by Maxwell–Garnett approximation (Figure 2C). In this approximation, the effective medium consists of a matrix medium with *ε_m_* and inclusions with *ε_i_*. The Maxwell–Garnett equation is [44]

(2) $$\frac{\varepsilon_{eff}-\varepsilon_{m}}{\varepsilon_{eff}+2\varepsilon_{m}}=\delta_{i}\frac{\varepsilon_{i}-\varepsilon_{m}}{\varepsilon_{i}+2\varepsilon_{m}},$$

where *ε_eff_* is the effective dielectric permittivity of the medium and *δ_i_* is the volume fraction of the inclusions. A comparison of the concentration dependence of the composites’ dielectric permittivity was obtained experimentally at 129 Hz and calculated according to Equation (2) with the parameters *ε_m_* = 4 (obtained experimentally) and *ε_i_* = 100 [45]. It can be concluded that the Maxwell–Garnet approximation describes the concentration behavior well.

**Figure 3:**
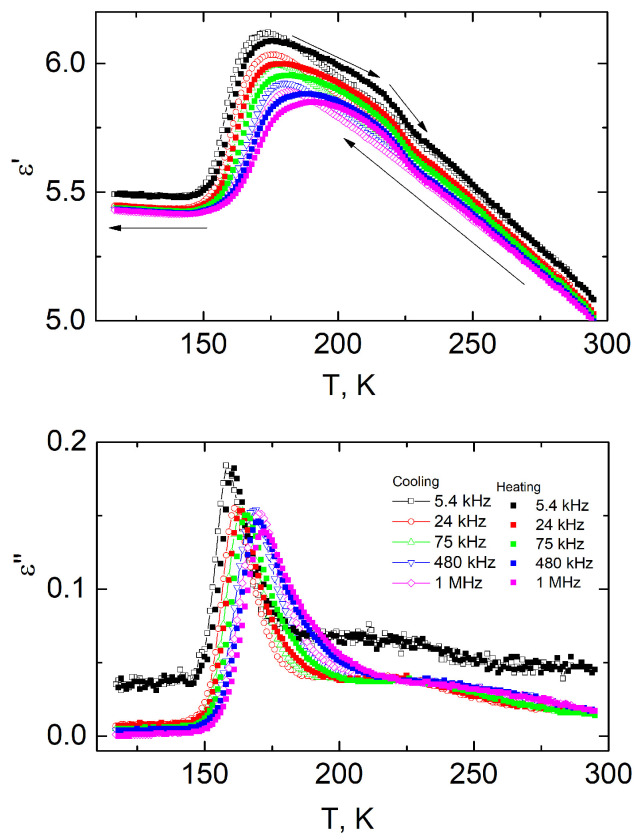
Temperature dependencies of the real and imaginary parts of the complex dielectric permittivity of PDMS filled with 10 vol.% TiO_2_ upon cooling and heating (arrows indicate the direction of temperature changes).

The temperature dependencies of the real and imaginary parts of the complex dielectric permittivity for composites containing 10 vol.% titanium dioxide are presented in Figure 3.

The temperature dependencies of the complex dielectric permittivity exhibit anomalous behavior around 180 K and 220 K. The position of the low-temperature maximum is frequency-dependent and is shifted to higher temperatures with increasing frequency. Similar dielectric properties are observed in pure PDMS and arise from large-scale segmental fluctuations corresponding to the dynamic glass transition (α-relaxation) of the polymer [46,47]. Above the glass transition and below the melting temperature, around 230 K, another peak is observed. This broad dispersion could be related to PDMS cold crystallization [48].

It is clearly observed that titanium dioxide nanoparticles affect the dielectric properties of the composites. The value of the dielectric permittivity increases significantly with the titanium dioxide concentration. Moreover, an increase in the real part of the complex dielectric permittivity at low temperatures, 150 K and below, is observed for high titanium dioxide concentrations (Figure 3) but not in pure PDMS.

**Figure 4:**
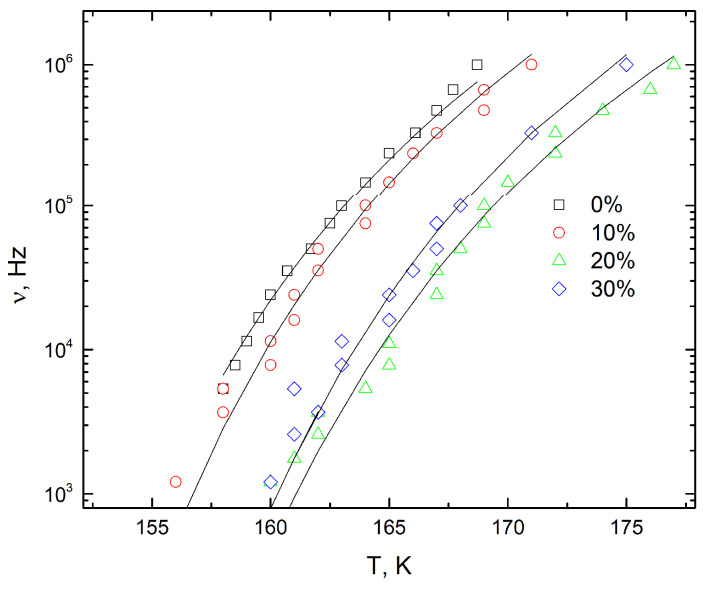
Measurement frequency as a function of temperature at maximum dielectric loss for PDMS composites with different volume concentrations of TiO_2_ indicated on the plot.

The maxima of the dielectric permittivity and losses as a function of temperature depend on the measurement frequency. The latter is presented as a function of the maximum dielectric loss position in Figure 4. The dependence was fitted using the Fogel–Vulcher relationship [49]:

(3) $$f=f_{0}e^{\frac{E}{k_{B}(T-T_{0})}}$$

where *k_B_* is the Boltzmann constant, *f*_0_ is the attempt frequency, *T*_0_ is the glass transition temperature of the polymer when the cooling rate becomes infinitely slow and E is the activation energy. The fit was performed using the same frequency value of *f*_0_ = 20 GHz for all the composites studied; the other parameters are presented in Table 1.

It can be concluded that the glass transition temperature increases with the nanoparticle concentration (for concentrations up to 30 vol.%) due to the increased coupling between the PDMS molecules close to the TiO_2_/PDMS interface. It should be admitted, however, that the reciprocal attempt frequency, (1/f), coincides with the relaxation time, *τ*, only if the distribution of relaxation times does not exist, i.e., when the dielectric dispersion can be described by the Debye equation:

(4) $$\varepsilon^{*}=\varepsilon_{\infty }+\frac{∆\varepsilon }{1+i\omega \tau },$$

where *ε*_∞_ is the dielectric permittivity at very high frequencies and Δ*ε* is the dielectric strength. However, if some distribution of the relaxation time *f*(*τ*) exists, it is related to complex dielectric permittivity ε* (*ω*) via an integral equation:

(5) $$\varepsilon^{*}(\omega )=\varepsilon_{\infty }+\Delta \varepsilon \int_{-\infty }^{\infty }\frac{f(\tau )dlog\tau }{1+i\omega \tau },$$

and in this case, the position of the complex dielectric permittivity ε*(*T*) maximum will be dependent on the frequency Δ*ε*(*T*), *ε*_∞_ (*T*) and distribution of the relaxation times *f*(*τ*).

### 3.2. Ultrasonic Investigations

To further investigate the influence of the titanium dioxide filler concentration on the glass transition and freeze–melt dynamics of the PDMS matrix, we performed ultrasonic measurements on the same composite samples. We started again with pure PDMS to obtain a reference response. Firstly, the longitudinal ultrasonic wave velocity, *V*, and attenuation, *α*, values were measured at room temperature. Next, temperature variations in terms of the signal amplitude and time of flight were measured upon cooling and subsequent heating to room temperature. These data enabled us to calculate the temperature dependencies of the attenuation, α, and relative ultrasonic velocity, δV/V=(V(T)−V)/V. No cooling–heating hysteresis was observed for the pure PDMS matrix. The anomalous temperature behavior of both δV and α for PDMS is centered around 180 K and exhibits a characteristic shape (not shown), usually observed when crossing the glass transition, in agreement with data published elsewhere and different scanning calorimetry investigations of pure PDMS [49,50].

**Figure 5:**
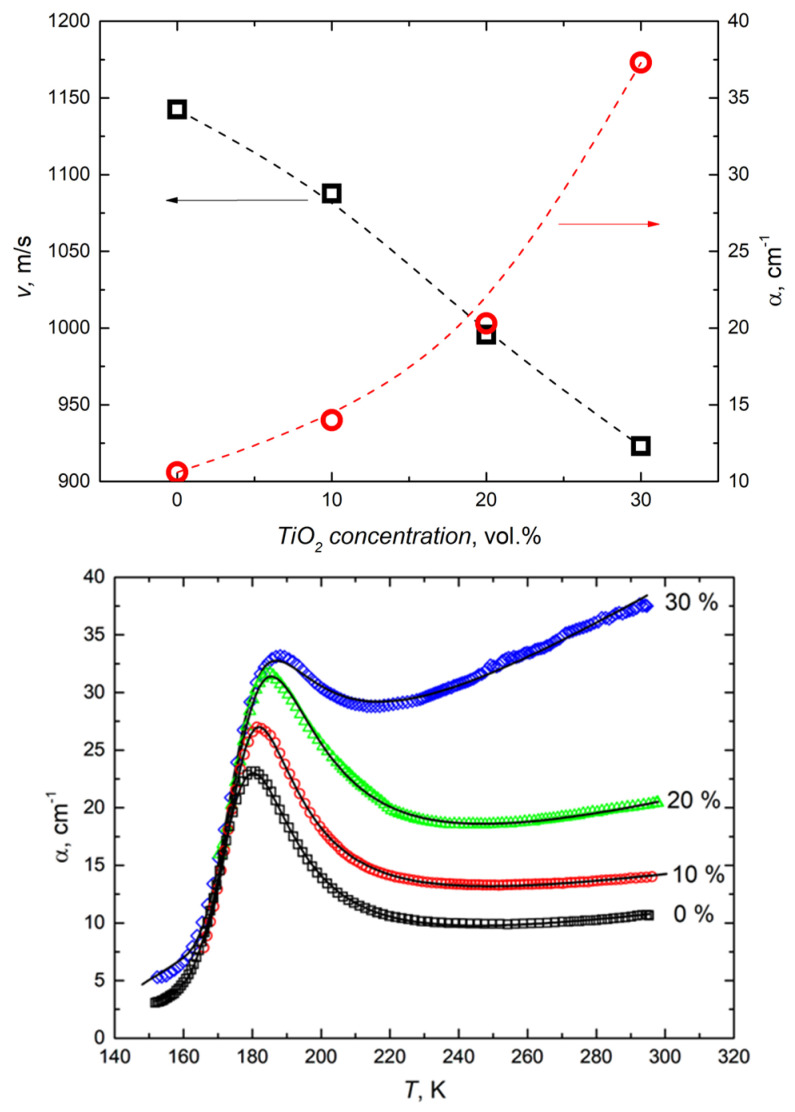
Filler concentration dependencies of ultrasonic velocity and attenuation at room temperature (**top**) and temperature dependencies of attenuation in composites with different filler concentrations (**bottom**).

The ultrasonic velocity and attenuation values obtained at room temperature, as well as the attenuation dependencies as a function of temperature for all the composites studied, are presented in Figure 5. The addition of titanium dioxide gradually reduces the ultrasonic velocity and increases the wave damping, as the nanoparticle inclusions act as additional scattering centers. The attenuation of ultrasonic waves upon cooling has its maximum close to 180 K. This maximum shifts to higher temperatures as the concentration of titanium dioxide nanoparticles increases. This peak can be described using the elastic relaxation theory and the assumption that for ultrasonic relaxation, the Debye type dispersion (no distribution of relaxation times) is observed [51]:

(6) $$\alpha =\frac{\Delta V}{V^{2}}\frac{\omega^{2}\tau_{u}}{1+\omega^{2}\tau_{u}^{2}}$$

where *V* and Δ*V* are, respectively, the longitudinal ultrasonic velocity in the composite and its magnitude of downward step at a certain temperature, *ω* is the angular frequency of the ultrasonic wave (10 MHz) and *τ_u_* is the relaxation time. We assume here that the relaxation time can be expressed by the Vogel–Fulcher equation:

(7) $$\tau =\tau_{0}e^{\frac{E}{k_{B}(T-T_{02})}}$$

where *τ*_0_ is the relaxation time when *T* → ∞. The obtained parameters are summarized in Table 2. The discrepancy between the *T*_0_ and *T*_02_ values in Table 1 and Table 2 is due to the fact that the dielectric dispersion is slightly broader than the Debye relaxation (Equation (4)). However, in both cases, the relaxation related to large-scale segmental fluctuations, i.e., *α* relaxation was investigated.

**Figure 6:**
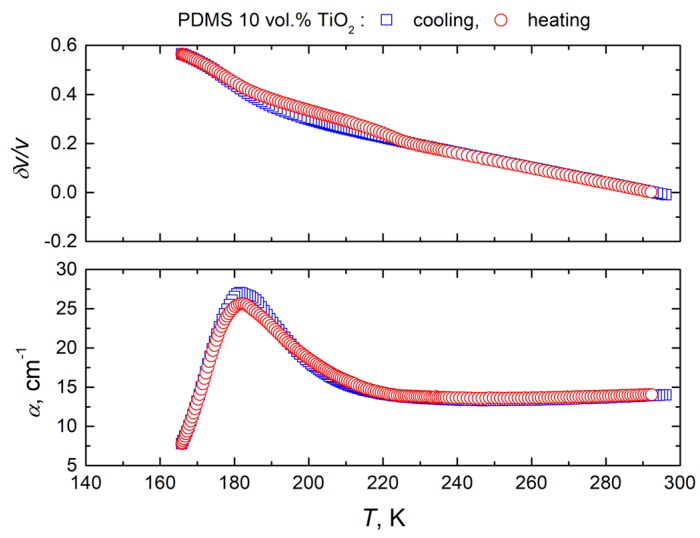

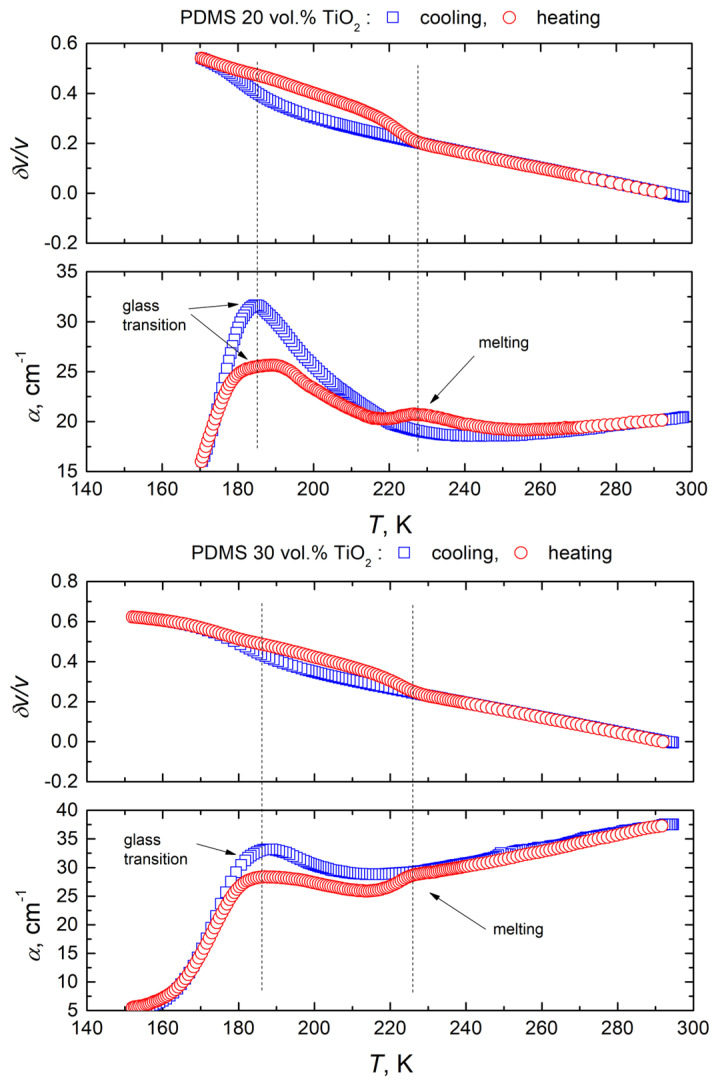
Temperature dependencies of ultrasonic velocity and attenuation at different concentrations of titanium dioxide nanoparticles upon heating and cooling.

The temperature dependencies of the ultrasonic velocity and attenuation at different concentrations of titanium dioxide nanoparticles upon heating and cooling are presented in Figure 6. The temperature hysteresis of these dependencies is caused by the crystallization/melt transition in the polymer matrix, which has already been investigated by various techniques, including differential scanning calorimetry in PDMS and PDMS-based composites [50,52]. Previous studies on PDMS composites with various fillers have shown that rigid nanoparticles can enhance crystallization and induce heterogeneous nucleation [49]. Titanium dioxide nanoparticles can act as local crystallization centers and therefore significantly enhance the absorption of ultrasonic waves upon heating. Moreover, it is clear that the ability to significantly influence the crystallization process depends on the distribution of nanoparticles in the polymer matrix. Poor distribution of titanium dioxide nanoparticles at higher concentrations is in good agreement with less pronounced hysteresis of the ultrasonic properties (Figure 1 and Figure 6). The linear increase in the ultrasonic attenuation coefficient with increasing temperature above 220 °C is related to the ultrasonic losses in the acoustic bonds and buffers (Figure 5 and Figure 6). Similar behavior was already observed in PDMS composites with zinc oxide nanoinclusions [49].

## 4. Conclusions

Broadband investigations of PDMS composites filled with titanium dioxide nanoparticles were performed in a wide temperature range, from 100 to 300 K. The dielectric permittivity was almost constant over the frequency range from 20 Hz to 3 THz at room temperature. The dielectric losses under these conditions were very low (less than 2), while the dielectric permittivity was rather high (up to 10 for composites with 30 vol.% inclusions). Therefore, these composites are suitable for various flexible electronic applications, for example, in a THz microfluidics platform [53]. These composites can also be used in high-efficiency multistimulus responsive actuators, where controlled dielectric properties are needed [54]. These composites can be used as additional layers in multilayer flexible sandwich structures for microwave and terahertz shielding applications [55]. In comparison with other dielectrics PDMS composites, they can be molded in various configurations and have good flexibility; thus, they have a wider range of applications [56]. They can also be used for microwave and terahertz lens preparation and as an effective substrate for flexible metamaterials, filters and modulators. It is important to note that the dielectric permittivity of these composites can be easily changed according to the Maxwell–Garnet model, which opens new possibilities for the application of these kinds of materials in various flexible electronics applications, particularly in the terahertz and microwave frequency ranges. The dielectric relaxation was only observed at lower temperatures (below 280 K) and at lower frequencies (below 1 MHz). This resulted from the relatively large-scale cooperative motion of many backbone segments in the amorphous phase of the PDMS in the bulk polymer and at the PDMS/TiO_2_ interface. The freezing temperature was substantially higher at the PDMS/TiO_2_ interface than in the bulk polymer volume, indicating strong interactions between the PDMS molecules and titanium dioxide nanoparticles. Ultrasonic studies have demonstrated that titanium dioxide nanoparticles act as a crystallization center and cause pronounced hysteresis in acoustic properties upon heating and cooling. Despite the fact that the activation energy values obtained from the dielectric and ultrasonic investigations were slightly different, these differences appeared due to the existence of distributions of the relaxation times in the PDMS matrix. Finally, the investigated composites can be used in flexible solar cells because titanium dioxide absorbs ultraviolet radiation and is transparent to visible light.

## Figures and Tables

**Table 1:** Parameters of the Vogel–Fulcher fit obtained from analysis of dielectric data (Equation (1)).

Concentration (vol.%)	*E*/*k_B_*, K	*T*_0_, K
0	343	135
10	331	137
20	371	139
30	341	140

**Table 2:** Parameters of the Vogel–Fulcher fit obtained from ultrasonic data analysis (Equations (2) and (3)).

Concentration	*τ*_0_, ns	*E*/*k*_B_, K	*T*_02_, K
0	1	208.9	133.6
10	2.3	139.2	144.6
20	2.9	161.3	139.3
30	8.3	85.8	150

## Data Availability

The original contributions presented in the study are included in the article, further inquiries can be directed to the corresponding author.
